# A Genome-Wide Association Study Identifies Quantitative Trait Loci Affecting Hematological Traits in *Camelus bactrianus*

**DOI:** 10.3390/ani10010096

**Published:** 2020-01-07

**Authors:** Fucheng Guo, Liang Ming, Rendalai Si, Li Yi, Jing He, Rimutu Ji

**Affiliations:** 1 Key Laboratory of Dairy Biotechnology and Bioengineering, Ministry of Education, College of Food Science and Engineering, Inner Mongolia Agricultural University, Hohhot 010018, China; guofucheng1101@163.com (F.G.); bmlimau@163.com (L.M.); sirendalai_imau@163.com (R.S.); yili_imau@163.com (L.Y.); hejing1409@163.com (J.H.); 2 Camel Research Institute of Inner Mongolia, Alxa 737300, China

**Keywords:** *Camelus bactrianus*, hematological traits, genome-wide association studies, loci

## Abstract

**Simple Summary:**

Bactrian camels can adapt to harsh natural environments. This unique tolerance of camels is tightly linked to their hematological traits, which are related to their immune, metabolic, and disease status. Therefore, mapping genomic regions that affect blood cell traits can help identify genomic characteristics that can be used as biomarkers of immune, metabolic, and disease states. This knowledge will further our understanding of the camel’s tolerance mechanisms.

**Abstract:**

Bactrian camels (*Camelus bactrianus*) are one of the few large livestock species that can survive in the Gobi Desert. Animal immunity and disease resistance are related to hematological traits, which are also associated with tolerance observed in Bactrian camels. However, no genome-wide association studies have examined the genetic mechanism of the immune capability of Bactrian camels. In the present study, we used genotyping-by-sequencing data generated from 366 Bactrian camel accessions to perform a genome-wide association study for 17 hematological traits. Of the 256,616 single-nucleotide polymorphisms (SNPs) obtained, 1,635 trait–SNP associations were among the top quantitative trait locus candidates. Lastly, 664 candidate genes associated with 13 blood traits were identified. The most significant were *ZNF772*, *MTX2*, *ESRRG*, *MEI4*, *IL11*, *FRMPD4*, *GABPA*, *NTF4*, *CRYBG3*, *ENPP5*, *COL16A1*, and *CD207*. The results of our genome-wide association study provide a list of significant SNPs and candidate genes, which offer valuable information for further dissection of the molecular mechanisms that regulate the camel’s hematological traits to ultimately reveal their tolerance mechanisms.

## 1. Introduction

Animal metabolism and immune status can be directly reflected by their hematological traits [1]. Therefore, screening for genomic regions that are associated with blood cell characteristics can identify genomic features that have a potential use as biomarkers to detect metabolic, immune, and disease status [2]. 

Camel species include the two-humped Bactrian camel (*Camelus bactrianus*) and the one-humped dromedary (*Camelus dromedarius*) [3,4]. China and Mongolia are the main countries that harbor Bactrian camels, and the absence of dromedaries in these locations [5,6] prevents hybridization between the two species. In addition, extant wild camels (*Camelus ferus*) are only distributed in these two countries [7,8]. *Camelus bactrianus* is one of the few large livestock animals that can survive in the Gobi Desert because of their ability to withstand the harsh natural environment. This tolerance is largely associated with their metabolic and immune states. Because the hematological characteristics of mammals reflect their immunity and disease resistance [9,10], deciphering the links between the blood traits and genes of camels will further our understanding of the molecular mechanisms by which these animals adapt to extreme environments. To date, many genome-wide association studies (GWASs) have sought causative mutations that affect the variation in hematological traits [11,12,13,14,15]. However, no GWASs have aimed to unravel the genetic mechanism that underlies the immune capability of *Camelus bactrianus*. 

A GWAS is a powerful approach for identifying genomic regions that control important zoological traits in non-structured germplasms [16,17]. A sufficient number of genome-wide markers can be produced using genotyping-by-sequencing (GBS), which is a high-throughput genotyping method that supports GWAS, even with limited species genomic information or tools [17,18,19,20]. Using clinical chemistry panels (CCPs) and complete blood count (CBC) tests, we performed comprehensive GWAS for 17 key hematological traits in the first comprehensive mapping study of the blood phenotypes of Bactrian camels.

## 2. Materials and Methods

### 2.1. Ethics Statement

The procedures and protocols for animal use were carried out according to the national codes of practice for the care and handling of farm animals and were approved by the animal care committee of the Camel Protection Association of Inner Mongolia.

### 2.2. Animals and Sample Collection 

This study included 366 domestic Bactrian camels. The sample population represented the seven main domestic *Camelus bactrianus* breeds in East Asia, including four Chinese domestic breeds (the Alxa Bactrian camel, the Junggar Bactrian camel, the Sunite Bactrian camel, and the Tarim Bactrian camel) and three Mongolian domestic breeds (the Galbiin Gobiin Ulaan Bactrian camel, the Haniin Hetsiin Huren Bactrian camel, and the Tokhom Tongalag Bactrian camel (see Supplementary Table S1)). Whole blood was collected from the jugular vein into vacutainers containing ethylenediaminetetraacetic acid (EDTA, for complete blood count tests, CBC) or no anticoagulant (for clinical chemistry panels, CCPs). All blood samples were analyzed within 24 h of sample collection. For CBC tests, the blood samples with the EDTA anticoagulant were analyzed using an automated hematology analyzer (ADVIA 2120, Siemens Healthcare Diagnostics Inc., Tarrytown, NJ, USA) with the manufacturer’s reagents and camel-specific settings. Analysis was carried out for total white blood cells (WBC), total red blood cells (RBC), average red blood cell volume (MCV), hemoglobin (HGB), mean hemoglobin content (MCH), hematocrit (HCT), total number of platelets (PLT), average platelet volume (MPV), mean hemoglobin concentration (MCHC), red blood cell distribution width-standard deviation (RDW_SD), red blood cell distribution width-coefficient of variation (RDW_CV), total platelet volume (PCT), and platelet distribution width (PDW). For the CCPs, the blood without the anticoagulant was centrifuged at 3000× *g* at 4 °C for 15 min. An automated chemistry analyzer (Modular P Chemistry Analyzer; Roche Diagnostics, Indianapolis, IN, USA) was used to analyze the separated serum using the manufacturer’s reagents. The analyzer calculated the levels of alanine aminotransferase (ALT), aspartate aminotransferase (AST), cholesterol (CHO), and glutamyl transpeptidase (GGT) (Table 1).

### 2.3. Genotyping

The sequence data were generated using genotyping-by-sequencing (GBS) [20,21]. The DNA was first digested using the methylation-sensitive restriction enzyme ApeKI (R0643L, New England Biolabs, Ipswich, MA, USA), and then the restriction fragments were ligated to a unique barcode adapter and universal adapter. The QIAquick PCR Purification Kit (28104, QIAGEN, Valencia, CA, USA) was used to purify an equal volume of pooled ligation products for PCR amplification. The amplicons were re-purified after amplification to generate clean PCR products. The generated library represented all 366 individual genotypes and was sequenced in each of the four lanes of an Illumina HiSequation PE150 instrument (Illumina, San Diego, CA, USA) to generate single-ended 100 bp reads.

The raw sequencing data were submitted to the National Center for Biotechnology Information (NCBI) Sequence Read Archive (SRA) in the GenBank database under accession number PRJNA565837.

### 2.4. Sequence Analyses and Single-Nucleotide Polymorphism (SNP) Calling

High-quality sequencing data were aligned to the *Camelus ferus* genome [9] using the BWA software [22] (parameters: aln -o 1 -m 100000 -t 4 -l 32 -i 15 -q 10). The reference genome address was downloaded from ftp://ftp.ncbi.nih.gov/genomes/Camelus_ferus/CHR_Un/. Population single-nucleotide polymorphism (SNPs) were detected using the SAMtools software [23]. Bayesian models were used to detect polymorphic loci in the population. SNP annotation was performed using the ANNOVAR software [24].

### 2.5. Estimating the Linkage Disequilibrium (LD) and Deducing the Population Structure

A phylogenetic tree and principal component analysis (PCA) were used to investigate the population structure. The TreeBeST software (http://treesoft.sourceforge.net/treebest.shtml) was used to calculate the distance matrix, which was the basis for constructing the phylogenetic tree using the neighbor-joining method. The distance between two bodies *i* and *j* was calculated using the following formula.

(1) $$D_{ij}=\frac{1}{L}\sum_{l=1}^{L}d_{ij}$$

where *L* is the length of the SNP region, and the allele at position 1 is A/C. If the genotypes of the two individuals are AA and AA, then *d_ij_* = 0. If they are AA and AC or AC and AC, then *d_ij_* = 0.5. If they are AA and CC, then *d_ij_* = 1.

The GCTA software (http://cnsgenomics.com/software/gcta/pca.html) was used to calculate the feature vectors and eigenvalues. The R software was used to draw the PCA distribution map. The SNP at position k in individual *i* is represented by *d_ik_*. If individual *i* is homozygous with the reference allele, then *d_ik_* = 0. If it is heterozygous, then *d_ik_* = 1. If individual *i* is homozygous with the non-reference allele, then *d_ik_* = 2. M is a matrix of *n* × *S* containing the standard genotype.

(2) $$d_{ik}=(d_{ik}-E(d_{k}))/\sqrt{E(d_{k})\times (1-E(d_{k})/2)/2}$$

The SNP data of the 366 camels were analyzed using the Frappe software to establish the population structure. First, we created an input file for software tool, PLINK, (http://pngu.mgh.harvard.edu/~purcell/plink/), a Ped file, and then used the Frappe software to construct the population genetic structure and group lineage information.

### 2.6. Statistical Analyses

The Bonferroni calibrated multiple test was used to determine the significance threshold, with a genomic significant level threshold of 0.05 per effective SNP locus and a chromosomal significant horizontal threshold of 1 per effective SNP locus. For the purpose of this study, the corresponding thresholds were set as 6.71 × 10^−6^ (0.05/256616).

### 2.7. Genome-Wide Association Study

GWAS analysis was performed using the GEMMA software (http://www.xzlab.org/software.html), and the population structure and individual kinship were corrected using the mixed linear model (MLM).

*y* = *Xα* + *Zβ* + *Wμ* + *e* (3)

where *y* is the phenotypic trait, *X* is the indicator matrix of the fixed effect, *α* is the estimated parameter of the fixed effect, *Z* is the indicator matrix of the SNP, *β* is the effect of the SNP, *W* is the indicator matrix of the random effect, *μ* is the predicted random individual, and *e* is a random residual and obeys *e*~(0, *δ_e_*^2^).

## 3. Results

### 3.1. Quality Assessment and Statistics of the Sequencing Data

Sequencing produced 548.54 Gb of data, with an average of 1.4987 Gb per sample, of which 548.52 Gb was high-quality clean data, with an average of 1.4986 Gb per sample (Supplementary Table S2).

### 3.2. Mapping to the Reference Genome

The sample rate was used to visualize the similarity between the sample sequencing data and the reference genome. The homology between the sequencing data and the reference sequence was evaluated by the coverage depth and coverage. The genome size was 2,009,177,929 bp, the average population ratio of the sample was 97.22–98.30%, the average genome sequencing depth ranged from 7.18× to 18.86×, and the 1× coverage rate (at least one base coverage) was above 6.46% (Supplementary Table S3).

### 3.3. SNP Calling and Annotation

At the genomic level, DNA sequence variations can be caused by an SNP, including single-base conversions and transversions. A total of 2,433,732 SNPs were identified using the SAMtools software, and the obtained SNPs were filtered (dp2, Miss0.5, Maf0.01). Lastly, 256,616 SNPs were obtained for subsequent analysis. The results of the ANNOVAR software analysis for SNP annotations are shown in Table 2. Among the SNPs, 1,193 were annotated as exonic and 70,370 as intronic. The intergenic area was annotated with the most SNPs (182,276).

### 3.4. Analysis of the Population Structure

**Figure 1:**
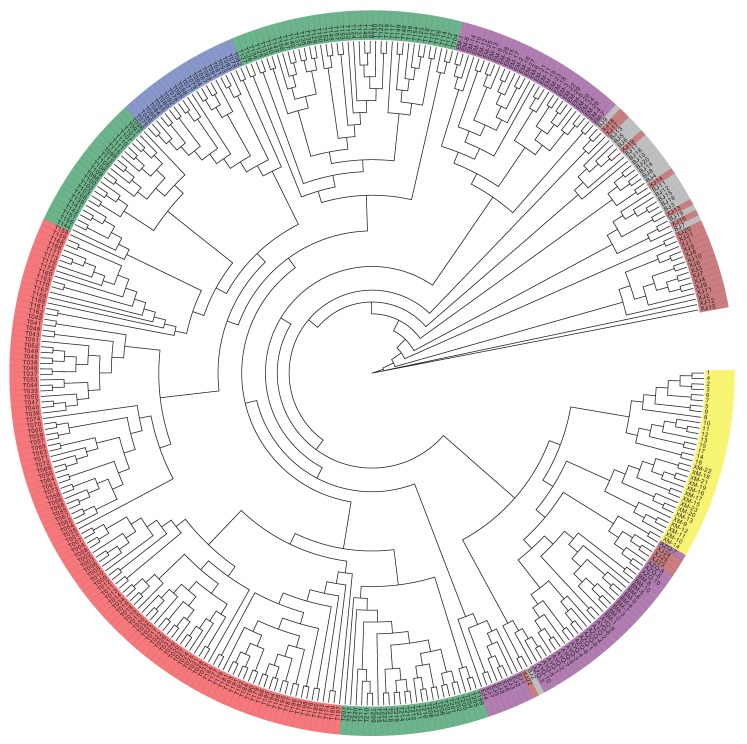
Systematic evolutionary tree of 366 Bactrian camels.

**Figure 2:**
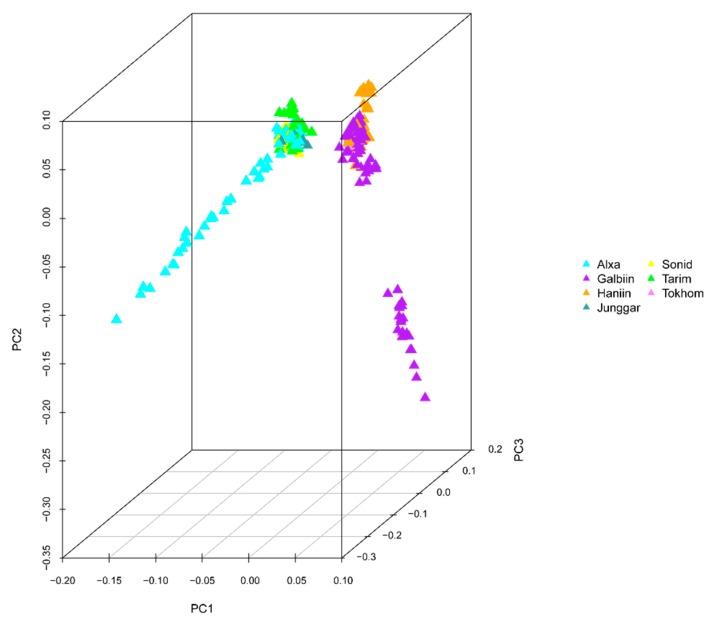
Principal component analysis (PCA) of 366 Bactrian camels. The sample population comprised four Chinese domestic breeds—the Alxa Bactrian camel (Alxa), the Junggar Bactrian camel (Junggar), the Sunite Bactrian camel (Sonid), and the Tarim Bactrian camel (Tarim)—and three Mongolian domestic breeds—the Galbiin Gobiin Ulaan Bactrian camel (Galbiin), the Haniin Hetsiin Huren Bactrian camel (Haniin), and the Tokhom Tongalag Bactrian camel (Tokhom).

The GWAS data were corrected for the presence of a population structure using three methods to identify relationships among the analyzed genotypes. The results of the phylogenetic tree showed that the 366 genotypes could be divided into seven groups, which is consistent with the number of breeds in the analysis (Figure 1). PCA results showed that four Chinese camel populations (Sunite, Alxa, Junggar, and Tari) and three Mongolian camel populations (Galbiin, Tokhom, and Haniin) were significantly separated at the PC1 level. However, the cross between the two groups was readily apparent, which indicates their close relationship (Figure 2). The population genetic structure usually refers to the non-random distribution of genetic variation in a population or species. According to their geographical distribution or other criteria, a group can be divided into several subgroups. Different individuals within the same subgroup have a closer kinship, while the relationships between individuals in different subgroups are more distant.

**Figure 3:**
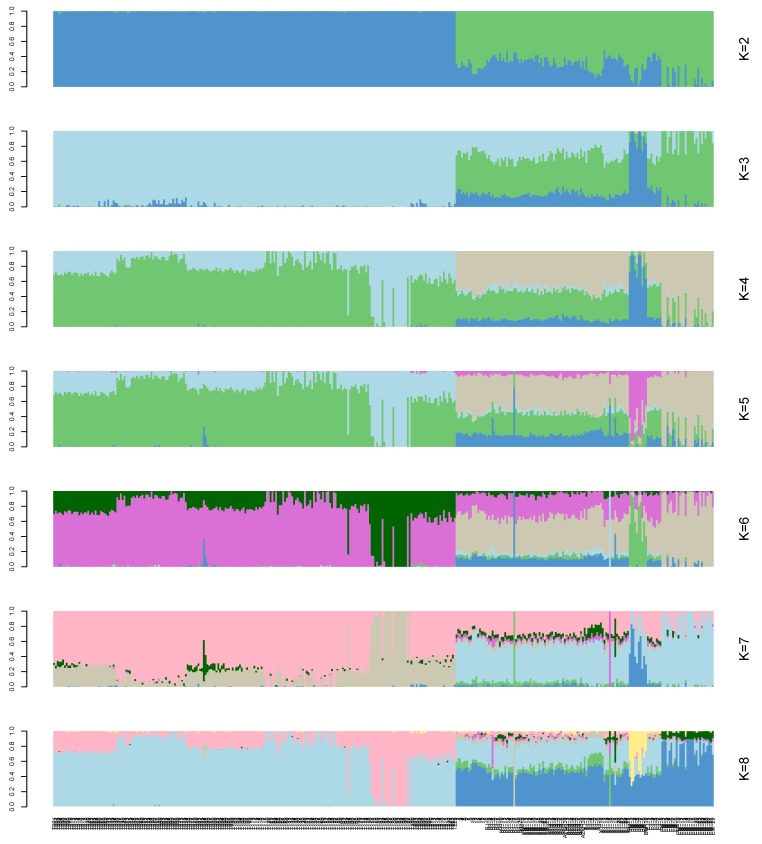
Population structure analysis of 366 Bactrian camels. The abscissa is each sample, and the ordinate represents the number of ancestors from 2 to 8.

A structural analysis was performed on the 366 samples. Each column in Figure 3 represents an individual, and the length of the different colored segments represents the proportion of an ancestor’s genome in the individual’s genome. K = 2–8 on the right side of Figure 3 indicates that 2–8 ancestral groups are assumed in this study. The abscissa of the picture indicates the name of the sample (the samples were sorted by group).

### 3.5. GWAS of Phenotypic Traits

**Figure 4:**
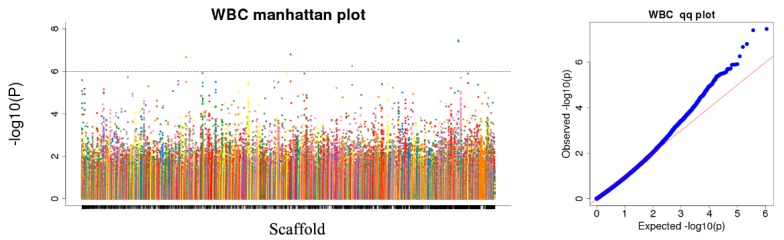
Manhattan plots and quantile–quantile (Q–Q) plots for white blood cell count (WBC). The left side of Figure 4 is the Manhattan map, which is the genetic marker effect value, i.e., the F-tested whole-genome *p*-value is sorted according to the physical position on the chromosome. The abscissa is the genomic coordinates, and the ordinate is −log10 (*p*-value). The smaller the *p*-value, the stronger the correlation, and the larger the ordinate. The horizontal dashed line in the Manhattan chart indicates the level of significance. When −log10 (*p*-value) > 6.26, the SNP is considered to be significantly associated with the trait.

We tested the association between each trait and the 256,616 SNPs. Quantile–quantile plot analysis showed that the model could reasonably control the false positive associations that potential population structures might cause. For the selected *p*-value of 10^−6^, the analysis identified 1,635 trait–SNP associations as the top quantitative trait loci (QTLs) (Supplementary Table S4). However, because of the multiple tests carried out, some of the reported associations might be false positives. The inter-chromosomal association patterns in the Manhattan plot show that trait-related SNPs are distributed throughout the genome (Figure 4, Supplementary Figures S1–S12).

To further evaluate the SNPs associated with the chosen traits, we aligned the tag sequence in which the SNP is located with all known sequences to infer its location and identify potential candidate genes that carry true etiological polymorphisms. The upstream and downstream 20 kb of 1635 SNP sites, which are known to be candidate genes in the camel genome, were searched randomly. Lastly, we identified 664 candidate genes associated with 13 blood traits (Supplementary Table S5, *p*-value < 10^−6^). WBC traits and RBC traits (MCV RBC, HCT, HGB, MCH, RDW_SD, RDW_CV), platelet traits (MPV, PCT, PDW), GGT, and CHO were identified as associated with 5, 226, 41, 50, and 342 genes, respectively. Table 3 lists the most significant candidate genes for the 13 blood traits according to gene function. None of the SNP markers showed associations with PLT, MCHC, AST, or ALT.

## 4. Discussion

In this study, we performed a GWAS for camel hematological traits using the genotypes of 366 domestic Bactrian camels from China and Mongolia. To the best of our knowledge, this is the first GWAS for camel hematological traits using GBS.

Population stratification affects the proportion of false positives in genome-wide association analysis. Therefore, corrections are needed to ensure the reliability of the statistical analysis [25,26,27]. Various strategies have been proposed to solve these GWAS-related problems [28,29], and we used three analyses to accurately determine the relationships among the analyzed genotypes. In the phylogenetic tree, the 366 genotypes are divided into seven groups, which is consistent with the number of breeds of the species in the study [7,30]. However, it is worth noting that a small number of Tarim Bactrian camel samples are mixed in with the Junggar Bactrian camel group, possibly because they all live in the Xinjiang region of China and hybridization events are inevitable. The PCA results distinguish the Chinese Bactrian camels from the Mongolian Bactrian camels very well. This clear distinction between breeds from different regions is likely the result of artificial constraints. Herders in both countries strictly restrict their camels’ active territory, so the animals are prevented from crossing national borders. The results of the structural analysis show that the Chinese Bactrian camels have more ancestral groups than the Mongolian Bactrian camels, which is likely to be related to the history of the Silk Road by which camels from other regions came to China [31]. Lastly, the quantile–quantile (Q–Q) plots show no evidence of the group stratification phenomenon, which demonstrates that the GWAS results based on the mixed linear model are relatively reliable.

The identification of markers that are tightly linked to phenotypic characteristics is the ultimate goal of genetic mapping [32,33]. The use of GBS generated a large number of genome-wide markers, which facilitated the GWAS in Bactrian camels. We identified 664 candidate genes associated with 13 blood traits. The most significant candidate genes according to gene function for the 13 blood traits are *ZNF772*, *MTX2*, *ESRRG*, *MEI4*, *IL11*, *FRMPD4*, *GABPA*, *NTF4*, *CRYBG3*, *ENPP5*, *COL16A1*, and *CD207* (for their full names, see Table 3).

WBCs are involved in the phagocytosis of bacteria and disease prevention. RBCs are essential for oxygen transport within the organism and the removal of the byproducts of respiration. The platelets play an important role in blood coagulation, wound healing, inflammatory response, and organ transplant rejection [34]. The white blood cell has emerged as a marker of inflammation that is widely available in clinical practice [35]. Studies have shown that zinc finger proteins are important regulators of white blood cell development, and the candidate gene *ZNF772* is likely to be involved in the regulation of early white blood cell differentiation [36]. Red blood cells are the most important components of oxygen transport through the blood in vertebrates, and they also have immune functions. Mtx2 is a protein in the mitochondrial sorting and assembly machinery (SAM) that has been implicated in TNF-induced apoptosis [37]. Gene ESRRG has been shown to be a negative regulator of anaerobic glycolysis [38] and might be related to energy metabolism in red blood cells, which affects the production of hemoglobin. The interaction of platelets with immune cells (neutrophils, monocytes, lymphocytes, and dendritic cells) can effectively enhance the function of immune cells. The candidate genes *CRYBG3* and *ENPP5*, as important protein structure regulators, are likely to be involved in the regulation of platelet volume and size.

Although we identified 12 candidate genes, their mechanism of action remains unclear and requires further study and confirmation. These candidate genes lay the foundation for further exploration of the characteristics of blood traits in Bactrian camels to reveal their tolerance mechanisms.

## 5. Conclusions

In conclusion, because blood traits are effective indicators of the camel’s immune and metabolic status, we attempted to identify genomic regions that control hematological traits in *Camelus bactrianus*. As a result, we identified 12 candidate genes and associated genetic markers, which provide a reference for further research on the camel’s special immune metabolism and disease resistance. Therefore, this study contributes to the understanding of the molecular regulation of blood traits in domestic animals.

## Figures and Tables

**Table 1:** Summary statistics of phenotypic traits used in genotyping-by-sequencing and the genome-wide association study (GBS-GWAS).

Trait	Mean	Standard Deviation	Minimum	Maximum
White blood cell count, 10^9^ cells/L (WBC)	21.95	11.07	4.01	65.5
Red blood cell count, 10^12^ cells/L (RBC)	8.64	2.11	4.8	28
Hemoglobin, g/L (HGB)	128.91	21.42	66	190
Mean corpuscular volume, fL (MCV)	41.83	4.67	34.5	58
Hematocrit, % (HCT)	0.35	0.13	0.16	1.4
Mean corpuscular hemoglobin, pg (MCH)	16.47	3.93	5.4	28.5
Mean corpuscular hemoglobin concentration, g/L (MCHC)	395	84.85	98	620
Red cell distribution width, % (RDW_SD)	56.72	7.1	45.1	91.3
Red cell distribution width, % (RDW_CV)	57.72	6.12	31.2	49.9
Platelet count, 10^9^ cells/L (PLT)	372.58	183.26	87.5	1007.5
Mean platelet volume, fL (MPV)	15.72	2.78	6.1	19.9
Plateletcrit, % (PCT)	0.58	0.33	0.11	1.98
Platelet distribution width, % (PDW)	0.16	0.06	0.11	0.25
Aspartate aminotransferase, U/L (AST)	105.5	25.15	58.8	185.8
Alanine aminotransferase, U/L (ALT)	15.43	4.13	25.4	4.2
Glutamyl transpeptidase, U/L (GGT)	25.53	12.69	6.5	83.2
Total cholesterol, mmol/L (CHO)	1.49	0.5	0.78	5.29

**Table 2:** SNP statistics and annotation results.

Items	Category	Number of SNPs
	Upstream	2671
Exonic	Stop gain	61
	Stop loss	5
	Synonymous	1380
	Non-synonymous	1496
	Intronic	155,104
	Splicing	37
	Downstream	3099
	upstream/downstream	28
	Intergenic	384,757
	ts	332,710
	tv	216,035
	ts/tv	1.540
	Total	548,745

Upstream: mutation in the 1 kb region upstream of the gene. Exonic: mutation in the exon region. Stop gain: mutation that causes the gene to acquire a stop codon. Stop loss: mutation that causes the gene to lose the stop codon. Synonymous: synonymous variation. Non-synonymous: non-synonymous variation. Intronic: mutation in the intron region. Splicing: mutation at the splice site (within 2 bp of the exon/intron boundary in the intron). Downstream: mutation in the 1 kb region downstream of the gene. Upstream/Downstream: mutation in the 1 kb region upstream of a gene and in the 1 kb region downstream of another gene. Intergenic: variation in the intergenic region. ts: transition. tv: transversion. Total: total number of SNP sites.

**Table 3:** Potential candidate genes identified by the genome-wide association study (GWAS) and based on genome annotations for the wild Bactrian camel.

Trait	Scaffold	Peak Position	Reference	alt	−log *p*-Value	Candidate Gene	Annotation
WBC	gi|558497537|ref|NW_006212822.1|	30,297	T	C	7.407936107	*ZNF772*	Zinc finger protein 772
RBC	gi|558499008|ref|NW_006211351.1|	31,481	C	T	12.64681147	*MTX2*	Metaxin 2
HGB	gi|558499111|ref|NW_006211248.1|	8,581,139	C	G	7.761062626	*ESRRG*	Estrogen-related receptor gamma
MCV	gi|558499592|ref|NW_006210767.1|	2,375,652	A	G	7.192791086	*MEI4*	Meiotic double-stranded break formation protein 4
HCT	gi|558499008|ref|NW_006211351.1|	31,481	C	T	11.84901895	*IL11*	Interleukin 11
MCH	gi|558498226|ref|NW_006212133.1|	120,804	T	G	6.462211621	*FRMPD4*	FERM and PDZ domain containing 4
RDW_SD	gi|558499308|ref|NW_006211051.1|	1,267,292	A	G	11.82658732	*GABPA*	GA binding protein transcription factor alpha subunit 60 kDa
RDW_CV	gi|558499308|ref|NW_006211051.1|	1,267,292	A	G	9.337752481	*GABPA*	GA binding protein transcription factor alpha subunit 60 kDa
MPV	gi|558500051|ref|NW_006210308.1|	893,732	T	A	9.240644582	*NTF4*	Neurotrophin-4
PCT	gi|558498489|ref|NW_006211870.1|	319,343	G	A	6.093058393	*CRYBG3*	Beta-gamma crystallin domain containing 3
PDW	gi|558498782|ref|NW_006211577.1|	5,532,111	T	G	14.12056347	*ENPP5*	Ectonucleotide pyrophosphatase/phosphodiesterase 5 (putative)
GGT	gi|558499927|ref|NW_006210432.1|	871,319	C	A	8.005790752	*COL16A1*	Collagen type XVI alpha 1
CHO	gi|558498607|ref|NW_006211752.1|	431,610	G	A	15.38590792	*CD207*	ATPase H^+^ transporting lysosomal 56/58kDa V1 subunit B1

## Supplementary Materials

The following are available online at https://www.mdpi.com/2076-2615/10/1/96/s1. Table S1: Sample information for 366 Bactrian camels. Table S2: Sequencing data statistics and quality assessment. Table S3: Sequencing depth and coverage statistics. Table S4: Genome-wide association study (GWAS)-related gene function annotation. Table S5: Candidate genes associated with blood traits. White blood cell, WBC. Red blood cell, RBC. Hemoglobin, HGB. Average red blood cell volume, MCV. Hematocrit, HCT. Mean hemoglobin content, MCH. Mean hemoglobin concentration, MCHC. Red blood cell distribution width SD, RDW_SD. Red blood cell distribution width cell volume (CV), RDW_CV. Average platelet volume, MPV. Total platelet volume, PCT. Platelet distribution width, PDW. Glutamyl transpeptidase, GGT. Cholesterol, CHO. Supplementary Figure S1: Manhattan plots and quantile–quantile (Q–Q) plots for the total number of red blood cells (RBCs). Supplementary Figure S2: Manhattan plots and quantile–quantile (Q–Q) plots for hemoglobin (HGB). Supplementary Figure S3: Manhattan plots and quantile–quantile (Q–Q) plots for the average red blood cell volume (MCV). Supplementary Figure S4: Manhattan plots and quantile–quantile (Q–Q) plots for the hematocrit (HCT) value. Supplementary Figure S5: Manhattan plots and quantile–quantile (Q–Q) plots for the mean hemoglobin content (MCH). Supplementary Figure S6: Manhattan plots and quantile–quantile (Q–Q) plots for the red blood cell distribution width SD (RDW_SD). Supplementary Figure S7: Manhattan plots and quantile–quantile (Q–Q) plots for the red blood cell distribution width cell volume (CV) (RDW_CV). Supplementary Figure S8: Manhattan plots and quantile–quantile (Q–Q) plots for the average platelet volume (MPV). Supplementary Figure S9: Manhattan plots and quantile–quantile (Q–Q) plots for the total platelet volume (PCT). Supplementary Figure S10: Manhattan plots and quantile–quantile (Q–Q) plots for the platelet distribution width (PDW). Supplementary Figure S11: Manhattan plots and quantile–quantile (Q–Q) plots for glutamyl transpeptidase (GGT). Supplementary Figure S12: Manhattan plots and quantile–quantile (Q–Q) plots for cholesterol (CHO).
