# Supplementary figures and images for: A Genome-Wide Association Study Identifies Quantitative Trait Loci Affecting Hematological Traits in Camelus bactrianus
Source: Animals (Basel). 2020 Jan 7;10(1):96. doi: 10.3390/ani10010096 (PMC7023321; doi:10.3390/ani10010096)

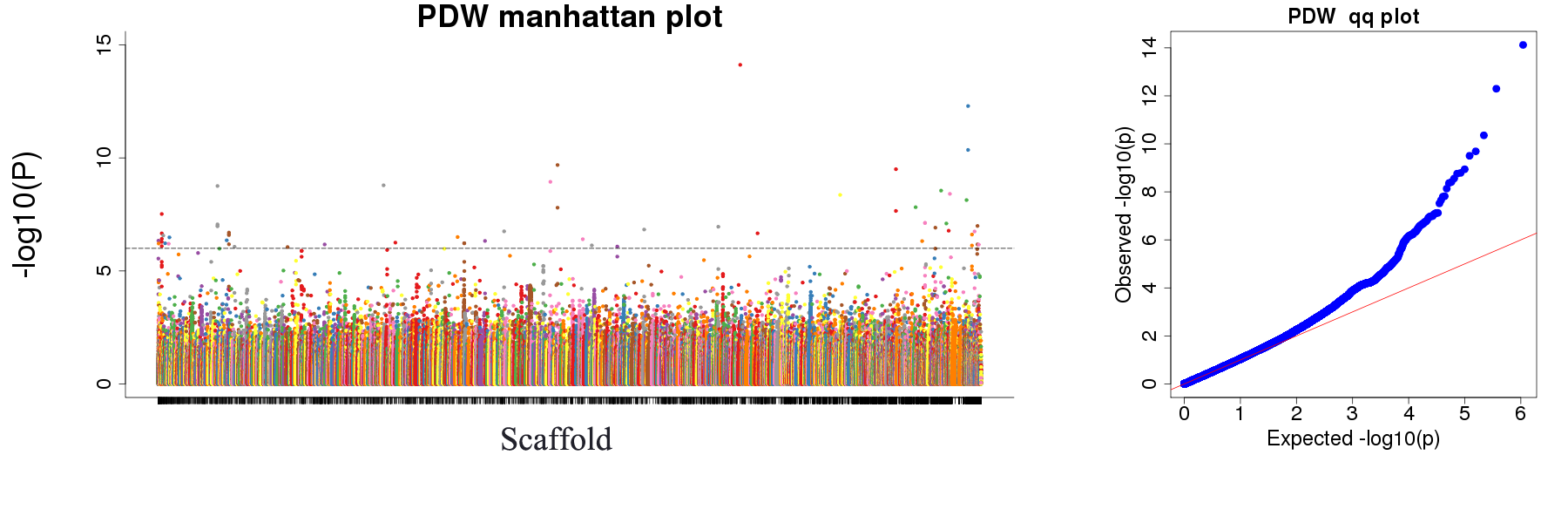

Supplement: Supplementary file 1 [file animals-10-00096-s001.zip › supplementary files/Supplementary Fig. 10_PDW.tif]

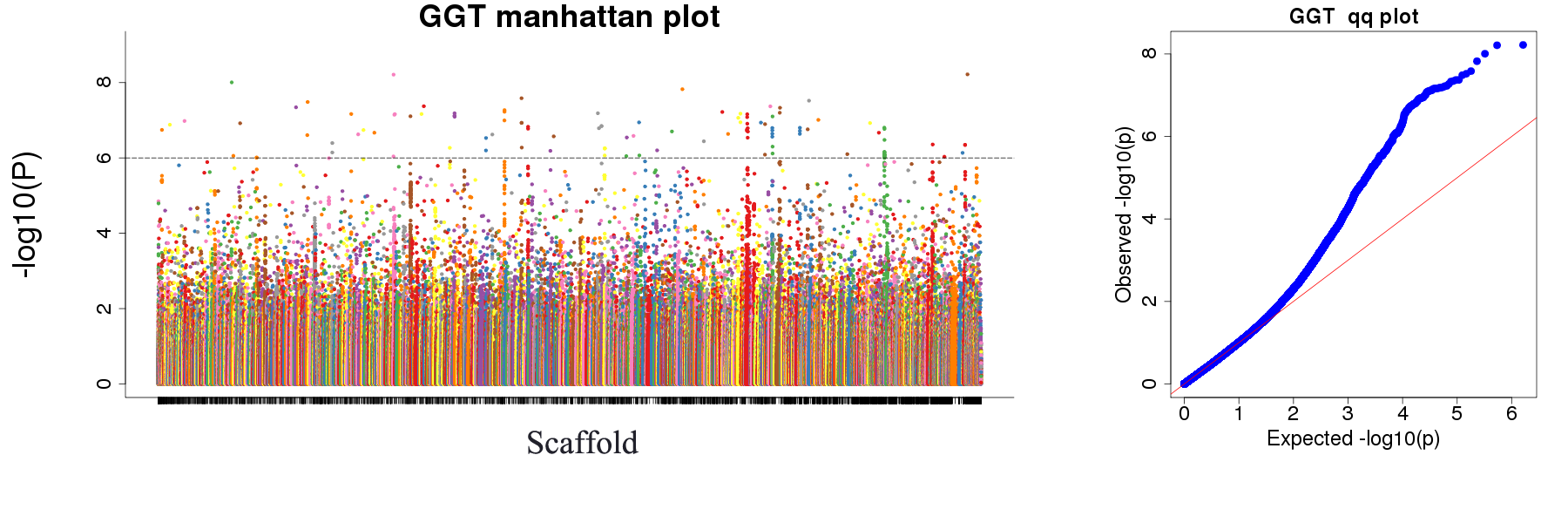

Supplement: Supplementary file 1 [file animals-10-00096-s001.zip › supplementary files/Supplementary Fig. 11_GGT.tif]

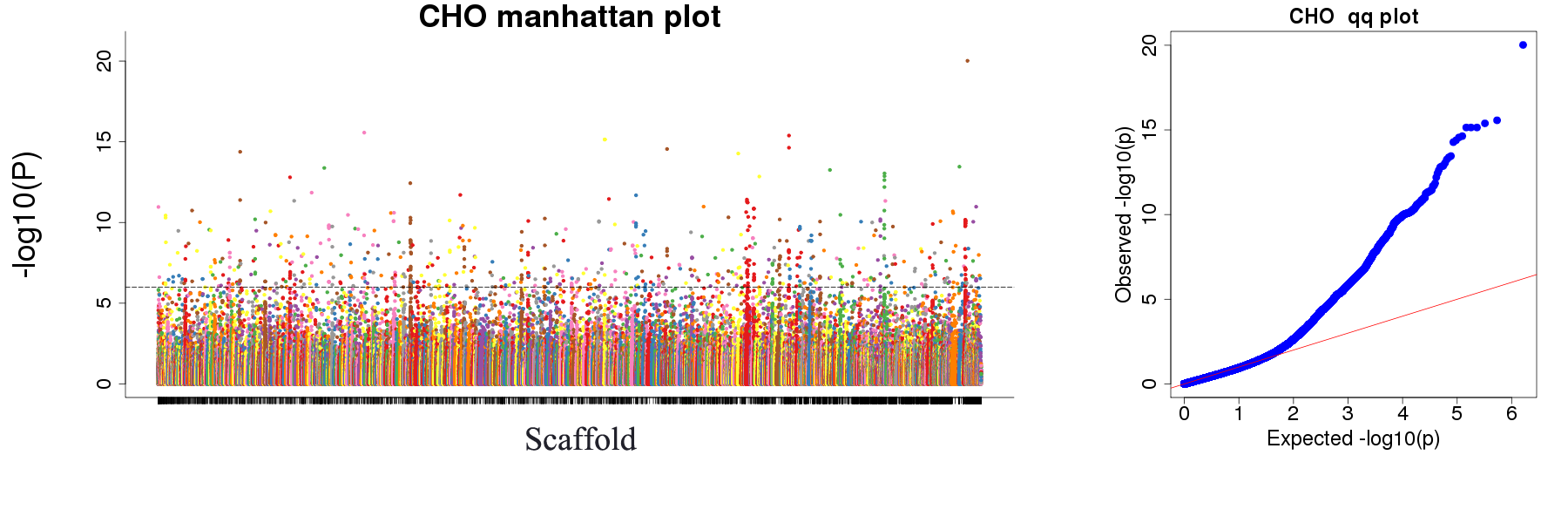

Supplement: Supplementary file 1 [file animals-10-00096-s001.zip › supplementary files/Supplementary Fig. 12_CHO.tif]

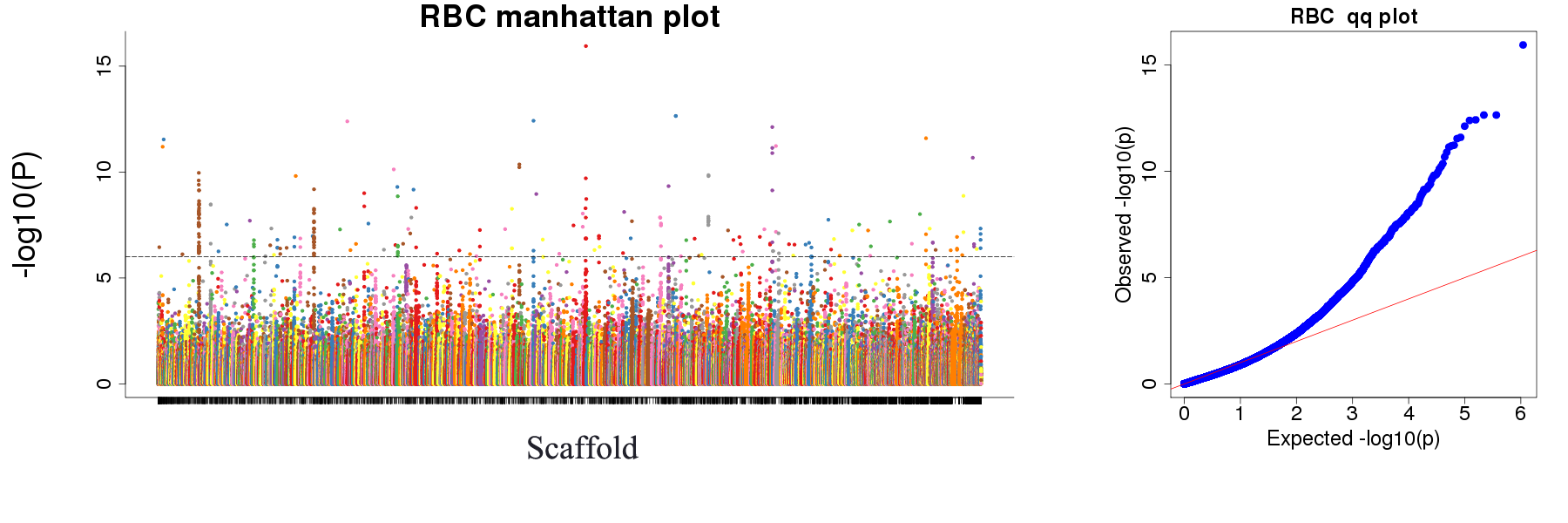

Supplement: Supplementary file 1 [file animals-10-00096-s001.zip › supplementary files/Supplementary Fig. 1_RBC.tif]

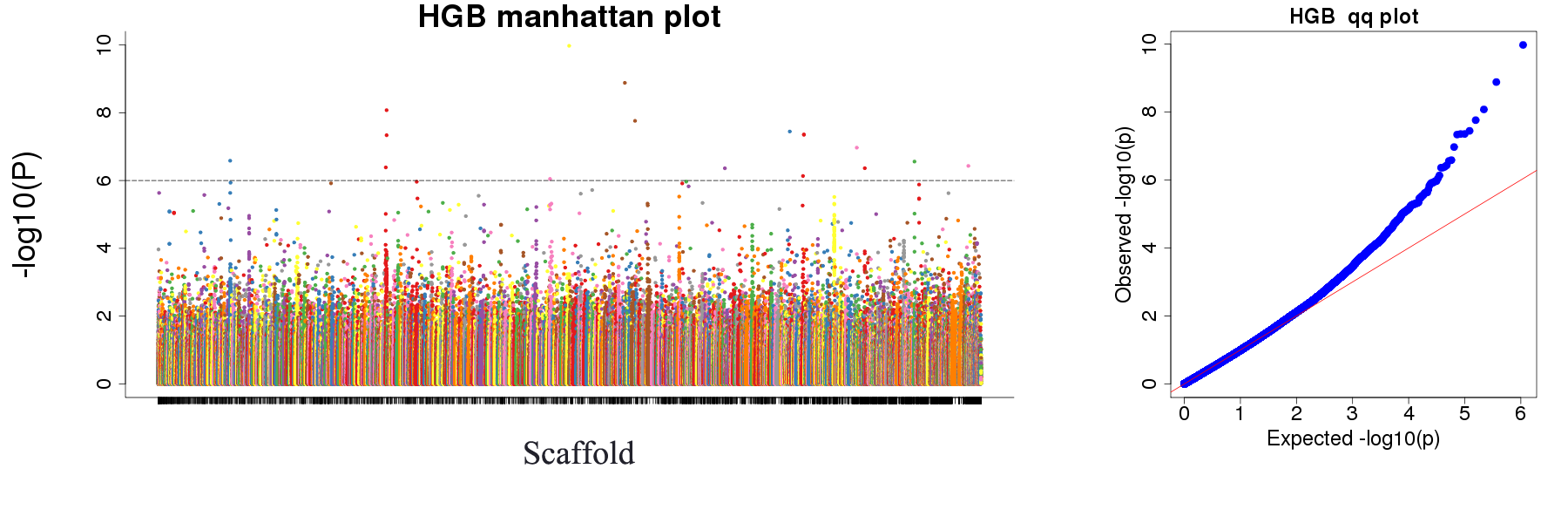

Supplement: Supplementary file 1 [file animals-10-00096-s001.zip › supplementary files/Supplementary Fig. 2_HGB.tif]

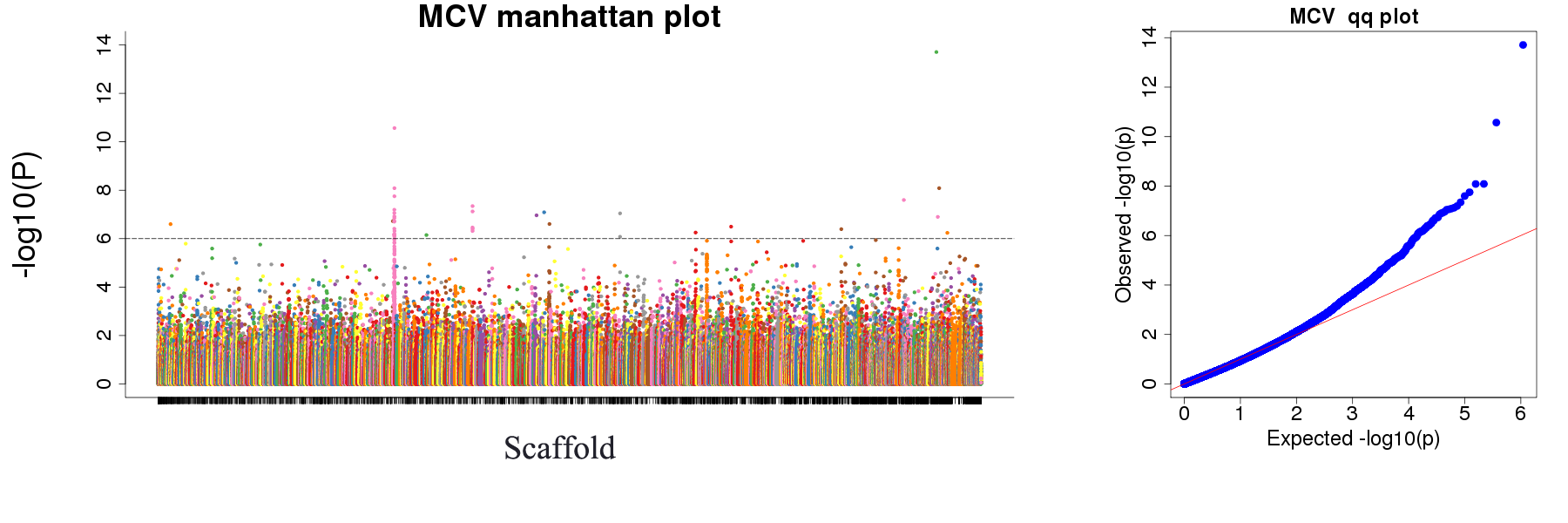

Supplement: Supplementary file 1 [file animals-10-00096-s001.zip › supplementary files/Supplementary Fig. 3_MCV.tif]

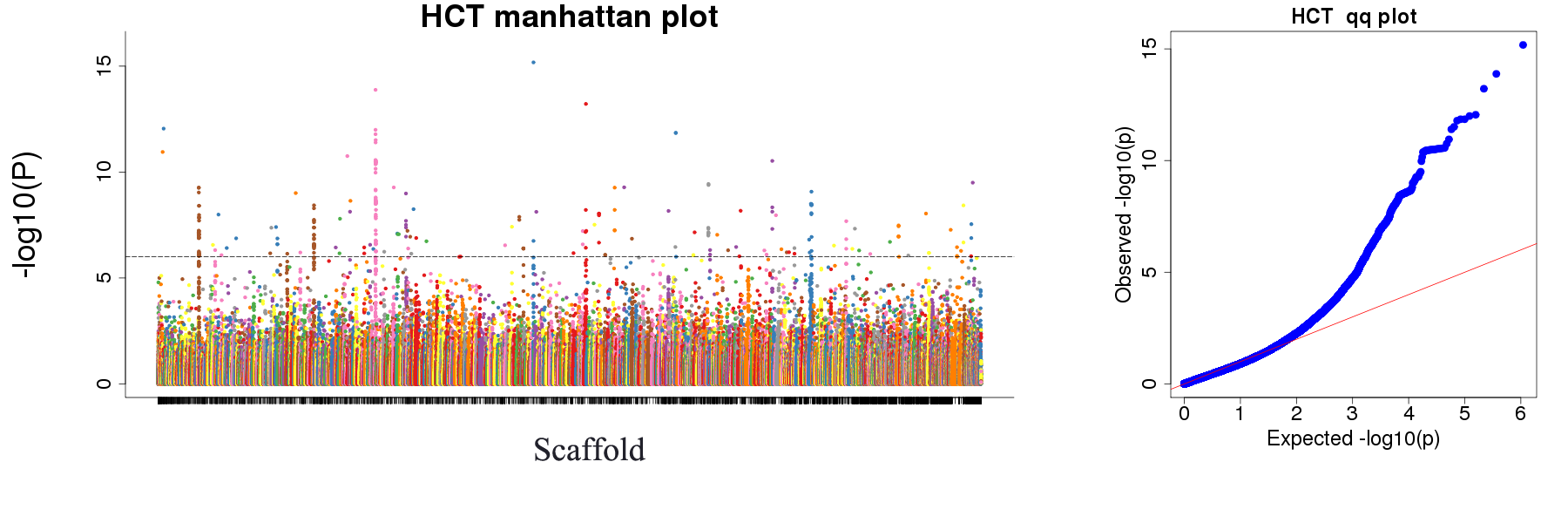

Supplement: Supplementary file 1 [file animals-10-00096-s001.zip › supplementary files/Supplementary Fig. 4_HCT.tif]

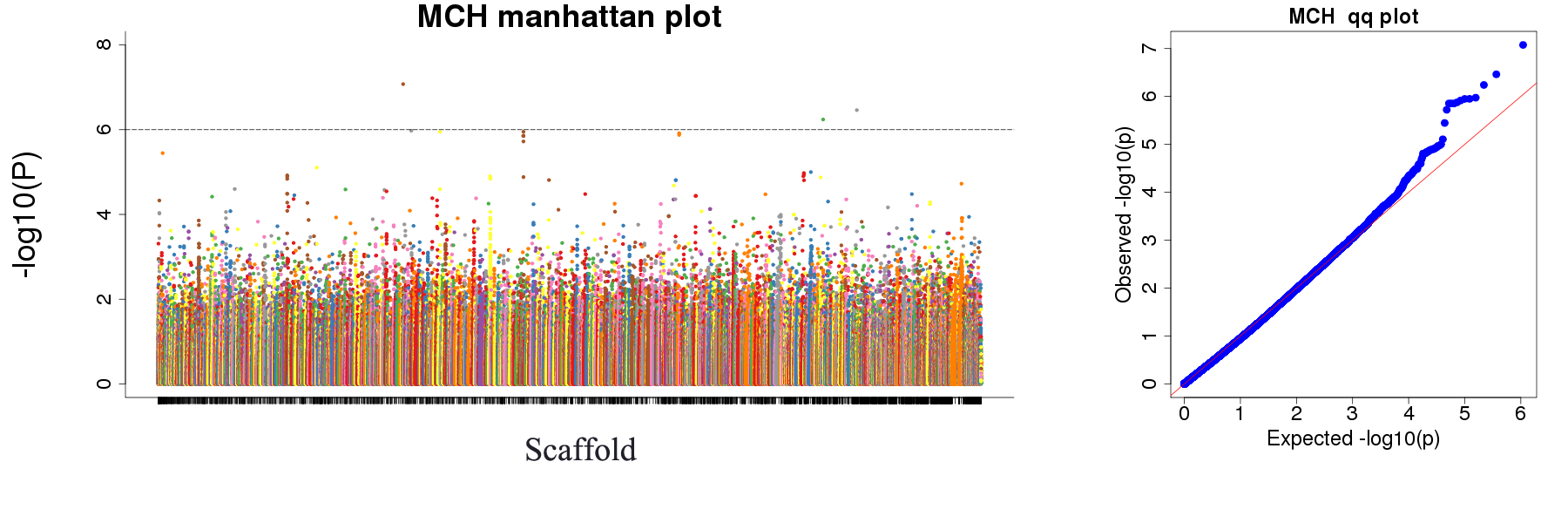

Supplement: Supplementary file 1 [file animals-10-00096-s001.zip › supplementary files/Supplementary Fig. 5_MCH.tif]

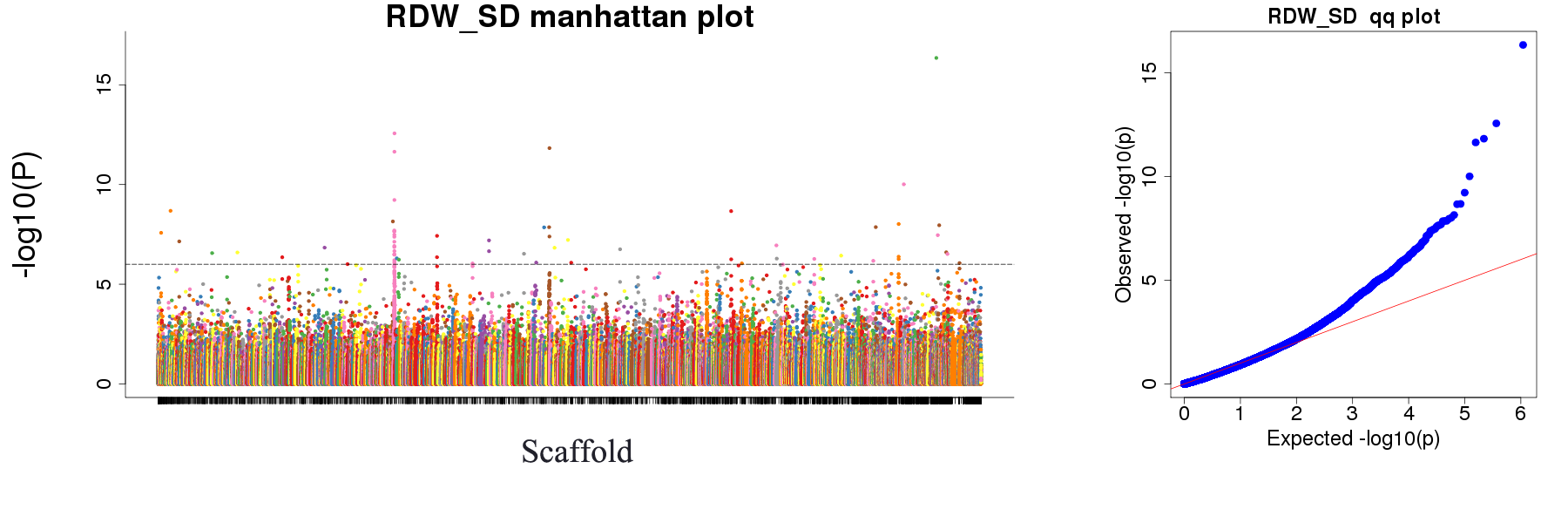

Supplement: Supplementary file 1 [file animals-10-00096-s001.zip › supplementary files/Supplementary Fig. 6_RDW_SD.tif]

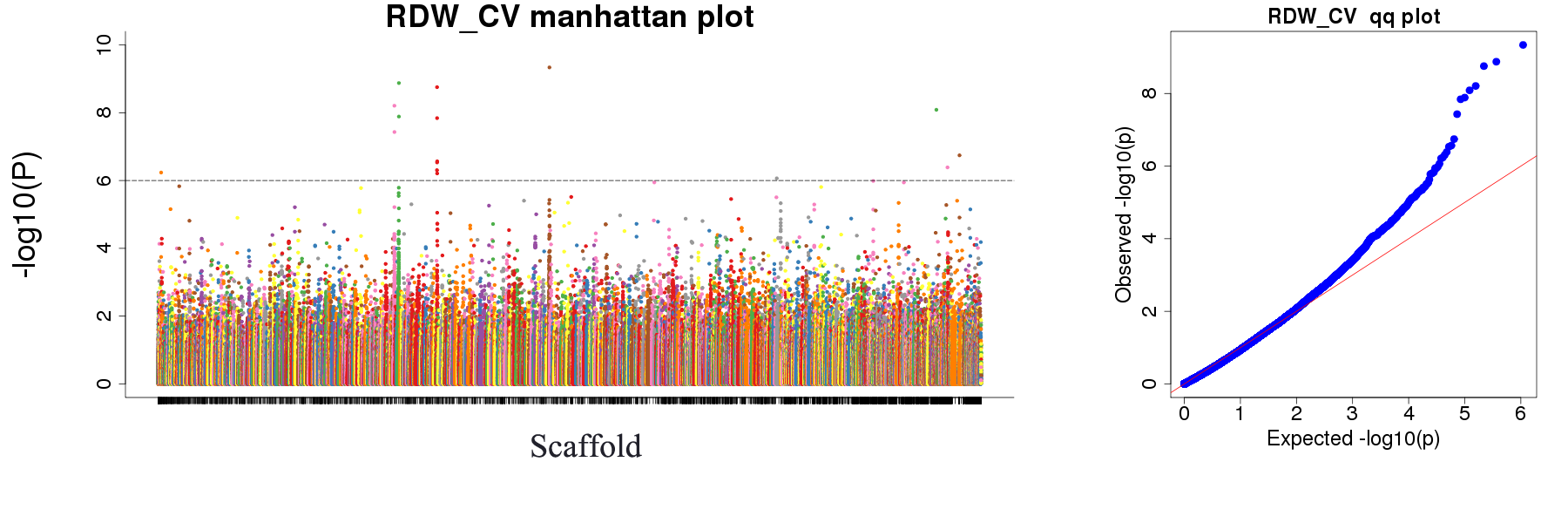

Supplement: Supplementary file 1 [file animals-10-00096-s001.zip › supplementary files/Supplementary Fig. 7_RDW_CV.tif]

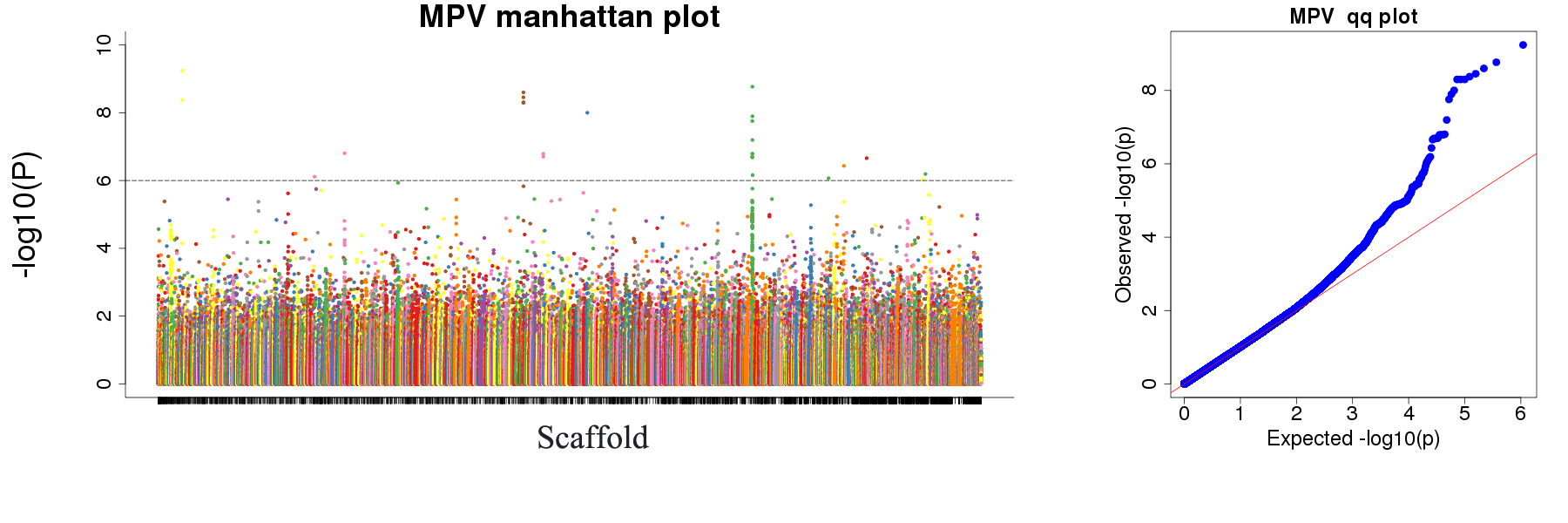

Supplement: Supplementary file 1 [file animals-10-00096-s001.zip › supplementary files/Supplementary Fig. 8_MPV.tif]

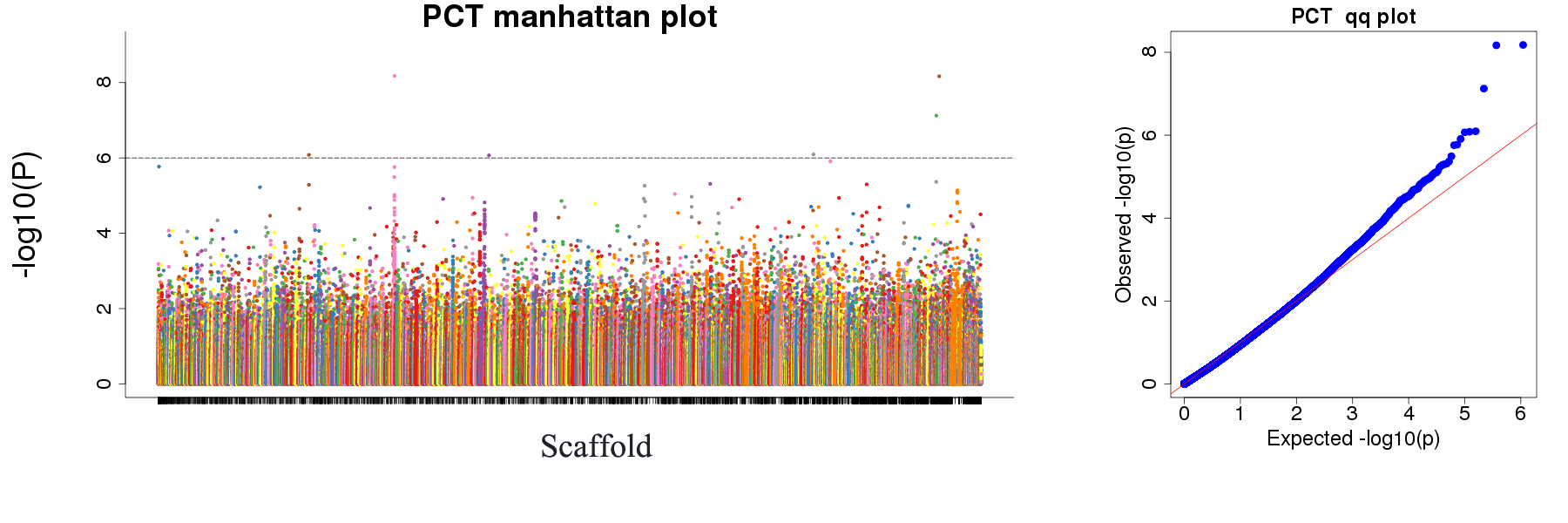

Supplement: Supplementary file 1 [file animals-10-00096-s001.zip › supplementary files/Supplementary Fig. 9_PCT.tif]
